# Aboriginal community controlled health organisations address health equity through action on the social determinants of health of Aboriginal and Torres Strait Islander peoples in Australia

**DOI:** 10.1186/s12889-020-09943-4

**Published:** 2020-12-04

**Authors:** O. Pearson, K. Schwartzkopff, A. Dawson, C. Hagger, A. Karagi, C. Davy, A. Brown, A. Braunack-Mayer, on behalf of the Leadership Group guiding the Centre for Research Excellence in Aboriginal Chronic Disease Knowledge Translation and Exchange (CREATE)

**Affiliations:** 1 Wardliparingga Aboriginal Health Equity Theme, South Australian Health and Medical Research Institute, Adelaide, Australia; 2 Faculty of Health and Medical Sciences, University of Adelaide, Adelaide, Australia; 3 Department of Community Health Sciences, University of Manitoba, Winnipeg, Canada; 4 Faculty of Social Sciences, University of Wollongong, Wollongong, Australia

**Keywords:** Social determinants of health, Aboriginal community-controlled primary health care, Indigenous, Primary health care

## Abstract

**Background:**

Indigenous populations globally are continually striving for better health and wellbeing due to experiencing significant health and social inequities. The social determinants of health are important contributors to health outcomes. Comprehensive primary health care that is governed and delivered by Indigenous people extends beyond the biomedical model of care to address the social determinants of health. Aboriginal Community Controlled Health Organisations (ACCHOs) are known to provide culturally informed, holistic health services that directly and indirectly address the social determinants of health. The range and extent of their activities in addressing the social determinants of health, however, is not well documented.

**Methods:**

The most recent ACCHO annual reports were retrieved online or by direct correspondence. For coding consistency, a dictionary informed by the World Health Organization’s *Conceptual Framework for Action on the Social Determinants of Health* was developed*.* A document and textual analysis of reports coded ACCHO activities and the determinants of health they addressed, including intermediary determinants, socio-economic position and/or socio-political context. Summary statistics were reported. Representative quotes illustrating the unique nature of ACCHO service provision in addressing the social determinants of health were used to contextualise the quantitative findings.

**Results:**

Sixty-seven annual reports were collected between 2017 and 2018. Programs were delivered to population groups across the life span. Fifty three percent of reports identified programs that included work at the socio-political level and all annual reports described working to improve socioeconomic position and intermediary determinants of health through their activities. Culture had a strong presence in program delivery and building social cohesion and social capital emerged as themes.

**Conclusions:**

This study provides evidence of the considerable efforts of the ACCHO sector, as a primary health care provider, in addressing the social determinants of health and health inequity experienced by Indigenous communities. For the Aboriginal and Torres Strait Islander population, ACCHOs not only have an essential role in addressing immediate healthcare needs but also invest in driving change in the more entrenched structural determinants of health. These are important actions that are likely to have an accumulative positive effect in closing the gap towards health equity.

**Supplementary Information:**

The online version contains supplementary material available at 10.1186/s12889-020-09943-4.

## Background

For many decades now, the World Health Organisation (WHO) has recognised that the health of individuals and populations is determined by more than just physical and biological factors but also the social, economic and political contexts in which people are born, live and grow [1]. These socio-economic and political contexts are operationalised through macro governance structures, policies and broad societal values that collectively shape the socio-economic positioning (SEP) of individuals and populations [2]. Together, the socio- political context and SEP impact on individual and population living and working conditions, behaviours, physical and social and emotional well-being. Collectively these are termed the Social Determinants of Health that directly and indirectly effect equity in health [2]. The social determinants of health are highly visible in relation to the health and wellbeing of Indigenous populations. Colonial structures and processes have created disparity across most political, social, health and economic outcomes experienced by Indigenous populations, compared to the dominant population [3]. At the macro socio-political level, policies continue to perpetuate and in some cases increase the level of inequity experienced by Indigenous populations [4]. This is evident in Australia, where the Indigenous population continues to experience poor health and social outcomes [5].

Indigenous Australians intimately understand that health is a complex integration and balance of physical, environmental, emotional, social, spiritual and cultural well-being. This complexity is not reflected within the Australian health care system. For example, culture is not often understood to be integral to health and wellbeing, even though it has been strongly linked to improved health outcomes [6]. Culture can be defined as “the set of distinctive spiritual, material, intellectual and emotional features of society or a social group … [which] encompasses, in addition to art and literature, lifestyles, ways of living together, value systems, traditions and beliefs’ [7]. Therefore, in order to deliver health services that provide holistic healthcare and embody Aboriginal culture, Aboriginal peoples have developed novel alternate models of primary health care.

In 1971 in Redfern Australia, the Aboriginal community, without government funding, launched an Aboriginal Medical Service to address long standing inequitable access to care that was available to non-Indigenous Australians [8]. This service set the precedent for the establishment of 147 Aboriginal Community Controlled Health Organisations (ACCHOs) across Australia and the peak representative body, the National Aboriginal Community Controlled Health Organisation (NACCHO) [9, 10]. Aboriginal Community Controlled Health Organisations, are ‘a primary health care service initiated and operated by the local Aboriginal community to deliver holistic, comprehensive, and culturally appropriate health care to the community who controls it, through a locally elected Board of Management’ [11]. This holistic view of health embraced by ACCHOs differs significantly from the dominant biomedical model of health care in Australia [12]. ACCHOs frequently support clients to tackle social factors such as racism, housing, income insecurity and employment. As Khoury [13] describes, ACCHOs ‘transcend the concept of a specialised medical clinic and function as community spaces through which Indigenous people attempt to deal with their immediate health needs and the underlying structural causes that produce very poor health outcomes’ (p.472).

Whilst the ACCHO sector is recognised for its provision of holistic and comprehensive primary health care [13, 14], the consistency, range and comprehensiveness of activities and services that address the social determinants of health are not well described [15]. This study was conceived by Indigenous leaders and executives from the ACCHO sector guiding the work of the Centre for Research Excellence in Aboriginal Chronic Disease Knowledge Translation and Exchange [16]. The Leadership Group highlighted an evidence gap regarding ACCHO service provision related to the social determinants of health. *The aim of this study, therefore, was to describe the range of services provided by ACCHOs that address the social determinants of health.*

## Methods

The Indigenous-led research team followed the principles of the *South Australian Aboriginal Health Research Accord* [17] and the National Health and Medical Research Council’s *Guidelines for Ethical Conduct in Aboriginal and Torres Strait Islander Health Research* [18]. Guidance was sought from ACCHO representative peak bodies in each jurisdiction and associated Aboriginal health ethics committees (or equivalent) regarding the need for ethical review for this analysis of publicly available documents. Most jurisdictions did not require an ethical review. In one state and one territory a full ethics submission was required and granted (see Acknowledgements). Ethics approval was not obtained from one state within the timeframe of the study and therefore ACCHOs within this state were not included.

The participants of interest included 122 ACCHO member services of the National Aboriginal Community Controlled Health Organisation [9]. Data was sourced from ACCHO Annual Reports for 2015/16 or the closest available report to the 2015/16 financial year. Annual reports typically document activities that an organisation has been engaged in for a one-year period. They were chosen since they provided sufficiently descriptive data and their inclusion in the study would not result in additional burden to ACCHOs. This was considered important in the context of a sector heavily burdened by funding and reporting requirements [19]. To ensure the sector was fully informed, all ACCHOs received an email from the research group describing the nature and purpose of the study.

To retrieve Annual Reports, we first searched ACCHO websites. Where reports could not be retrieved using this method, ACCHOs were contacted via email which included a letter explaining the study and inviting the service to provide an electronic or hardcopy of their 2015/16 (or latest available) annual report. Additionally, 70 ACCHOs received a phone call from the research team to invite participation in the study. Data retrieval was conducted over 9 months (May 2017 to January 2018).

### Analysis and reporting framework

**Fig. 1:**
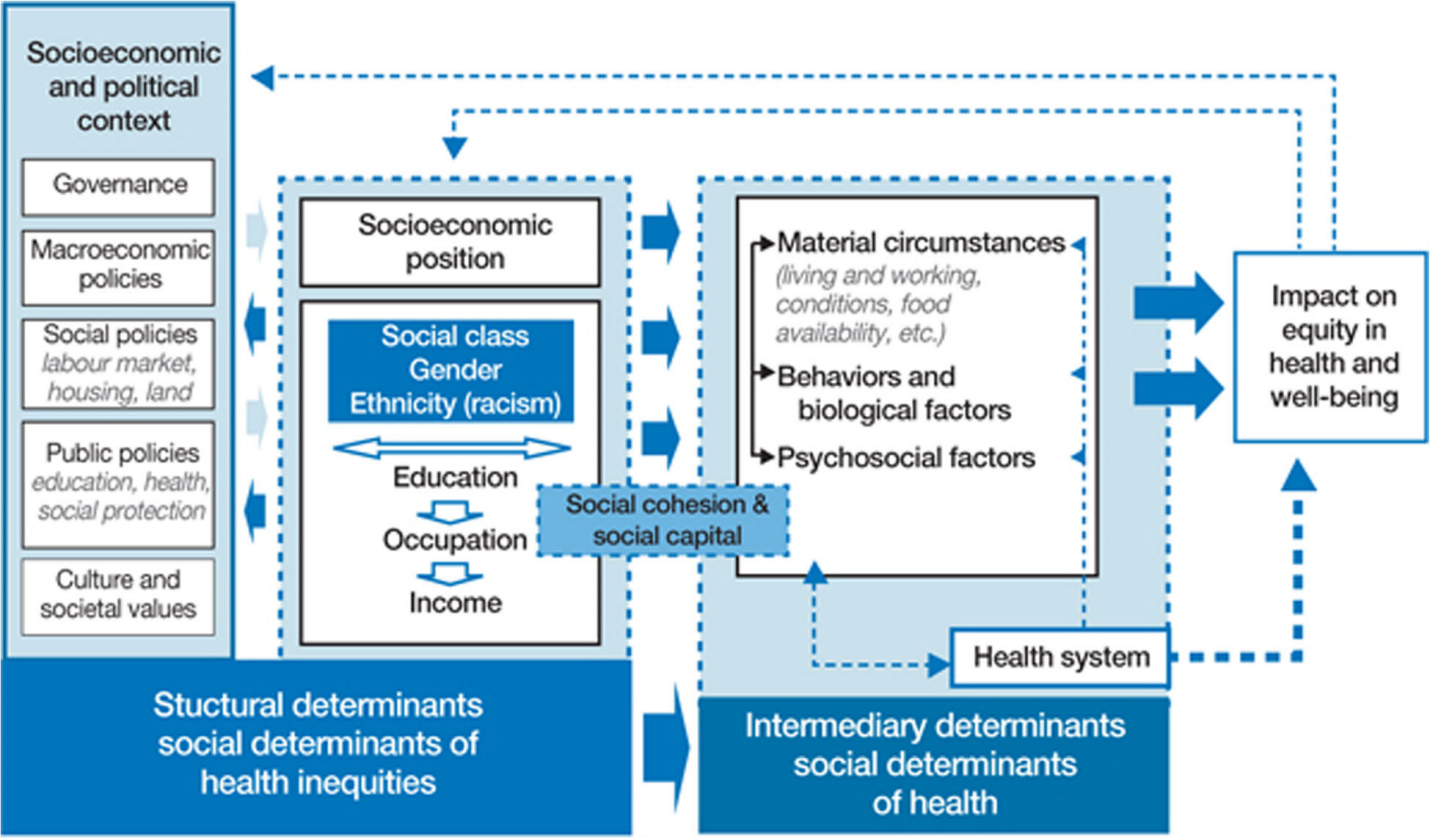
Conceptual Framework for the Social Determinants of Health (reproduced with permission)

The World Health Organization (WHO) developed a *Conceptual Framework for Action on the Social Determinants of Health* (Fig. 1) [2]. This was to enable a better understanding of the complexities of health within societal structures, and with the aim of assisting governments and public health organisations to address the social determinants of health to strengthen health equity. This study applied the WHO Framework to guide the analysis and report the work ACCHOs are doing to address the social determinants of health impacting Aboriginal and Torres Strait Islander people.

Specifically, data was analysed and reported in three categories:

The *socio-economic and political context* included the work ACCHOs reported that directed, developed, informed or advocated for improved political and government structures or policies including of a social, public or economic nature and work that aimed to influence cultural and societal values towards greater understanding, acceptance and inclusion of Aboriginal and Torres Strait Islander people;

*Socio-economic position*, included the work ACCHOs reported in their annual report that connected, provided or supported individuals, families or communities related to education, occupation and income and to reduce racism; and.

*Intermediary determinants*, perhaps the most familiar social determinants of health, included the work ACCHOs reported that addressed material circumstances (e.g., housing, employment, food subsidies), biological and behavioural factors (e.g. physical exercise, smoking and diet) or psychosocial factors (e.g., life events, social support).

Document and thematic analyses were conducted and summary statistics reported. Document analysis is an iterative, non-intrusive process applied to existing text to enable analysis to seek new meaning or answer research questions, and is performed via a *‘systematic and enumerative approach’* (pg. 80) [20]. A thematic analysis identified overarching and emerging themes related to the reported ACCHO ways of working.

Annual Reports were imported into NVivo 11 Pro (QSR International). A Coding Dictionary was developed by the Research Team to ensure coding consistency, based on the team’s knowledge of ACCHO service provision and the World Health Organization’s *Conceptual Framework for Action on the Social Determinants of Health* [2] (Fig. 1). Data was coded by one member of the research team due to feasibility restraints. However, together the research team developed a comprehensive coding dictionary to help achieve a level of coding consistency. Specifically, services described within ACCHO Annual Reports were coded to activities defined in the dictionary and then categorised into the area of focus; either client activities or activities for workforce. For each activity, where possible, the target population was identified. Furthermore, whether the activity addressed the socio-political context, socioeconomic position and/or intermediary determinant of health was recorded. Examples that showcased the unique nature of ACCHO service provision were identified during the coding process and representative quotes were presented in the results. Where information in quotes was deemed identifiable, the text was replaced with broad descriptors such as ‘[local]’ or ‘[local service]’.

Summary statistics included count and proportion of reports retrieved by location, method of retrieval (Table 1), activities, target population and determinants of health addressed (Table 2). Location was classified using the Australian Statistical Geography Standard Classification (ASGSC) [21].

**Table 1:** ACCHO Annual Reports, Retrieval Methods and Geographical Distribution

	***ACT***	***NSW***	***NT***	***QLD***	***SA***	***TAS***	***VIC***	***Total (%)***
*Total ACCHOs*^a^	*1*	*40*	*20*	*25*	*12*	*1*	*23*	*122*
*Annual Reports Retrieved (%)*	*1 (100%)*	*13 (33%)*	*13 (65%)*	*15 (60%)*	*10 (83%)*	*1 (100%)*	*14 (61%)*	*67 (47%)*
***Retrieval Method***								
*Online*	*1*	*11*	*13*	*14*	*6*	*1*	*11*	*57*
*Email/Letter*	*0*	*0*	*0*	*0*	*0*	*0*	*2*	*2*
*Phone Call*	*0*	*2*	*0*	*1*	*4*	*0*	*1*	*8*
***Geographical Distribution***								
*Major City*	*1*	*2*	*0*	*5*	*3*	*0*	*4*	*15 (83%)*^b^
*Inner Regional*	*0*	*7*	*0*	*3*	*1*	*1*	*4*	*16 (39%)*
*Outer Regional*	*0*	*2*	*1*	*5*	*2*	*0*	*6*	*16 (27%)*^b^
*Remote*	*0*	*2*	*7*	*2*	*2*	*0*	*0*	*13 (48%)*^b^
*Very Remote*	*0*	*0*	*5*	*0*	*2*	*0*	*0*	*7 (30%)*^b^

Notes: Geographical classification according to the Australian Standard Geographical Classification (ASGC) [21]

[Table 1 Legend: A description of the participating ACCHOs classified by State, method of retrieval and geographical distribution].

^a^as per NACCHO Member list as of 27/10/2017

^b^ACCHO reports retrieved as a proportion of total ACCHOs within the geographical area

**Table 2:** Determinants of health addressed by ACCHO activities reported

*ACCHO Activities*	*Total coded annual reports N(%)*	*Structural determinants: social determinants of health inequities*		*Intermediary determinants: social determinants of health*
		***Socio- political context***	***Socioeconomic position***	
*Client activities*^a^				
Total ANNUAL REPORTS CODED n(%)	**67 (100)**	**36 (53.7)**	**67 (100)**	**67 (100)**
Clinical Care	62 (92.5)	0	0	62
Community and Cultural Engagement	59 (88.1)	1	27	55
Health Promotion	59 (88.1)	1	20	58
Drug, Alcohol and Addiction	53 (79.1)	2	8	50
Mental Health	52 (77.6)	2	8	50
Family Support	48 (71.6)	1	10	41
Schools, Education and Training	47 (70.1)	1	37	23
Case Management	45 (67.2)	1	4	40
Advocacy	42 (62.7)	33	15	29
Transport	37 (55.2)	0	6	32
Personal Empowerment	37 (55.2)	0	9	27
Capacity Building and Community Empowerment	31 (46.3)	5	16	24
Legal and Justice Services	28 (41.8)	5	2	27
Financial Services	26 (38.8)	0	6	24
Aging and disability CARE^b^	22 (32.8)	0	3	21
Housing and Homelessness	22 (32.8)	2	2	21
Employment	15 (22.4)	0	11	4
*WORKFORCE AND Partnership Activities*				
Workforce	67 (100)	0	67	27
Collaborations & partnerships	57 (85.1)	13	31	48
Workforce training	51 (76.1)	1	52	20
Building Cultural Competency	33 (49.3)	0	31	15

[Table 2 Legend: The social determinants of health and health inequity addressed by ACCHOs through the range of services they provided.]

^a^ activities were coded to all applicable social determinants categories and therefore could be found to address one or more areas of the WHO framework and can result in totals adding to more than 67. For example, an income or employment program may have also added addressed housing and would be coded to both Socio-economic Position and Intermediary Determinants

^b^ in-community programs for older people and people with a disability to assist them to continue to live independently

## Results

Sixty-seven annual reports were analysed, representing 55% of the reports we sought to retrieve. Reports were retrieved through online searches (85%), phone calls (12%) and email correspondence (3%) over the 9-month period (May 2017 to January 2018). The sample included reports predominantly from 2015/2016 (60%), as per our search strategy, though also from other years where that financial year was the latest report available (2016/17 (21%), 2014/15 (13%), 2013/14 (5%)) (Table 1). ACCHOs described activities that were delivered to a range of population groups, namely: Mothers and Babies; Children and Young People; Women; Men; Older People; Families; People with Disability; People in the justice system; and LGBTQI people (Additional file 1) ACCHOs activities were extensive and included an abundance of programs for clients but also activities that targeted the ACCHO workforce and extended to local partnerships. The *Client Activities* and *Workforce and Partnership Activities* described by ACCHOs are presented in Table 2 in descending order of frequency, along with the WHO Framework determinants of health (intermediary, socioeconomic and socio-political contexts) they were thought to address.

Many activities that ACCHOs engaged in could not be categorised into one area of the WHO Framework since they often addressed community need using a multifaceted approach. So, while they may address the more urgent intermediary determinants, ACCHOs also worked to improve the structural determinants that impacted their clients and communities. As a result, activities were coded to all relevant determinant of health categories.

### Socio-economic and political context

The analysis revealed that ACCHOs addressed the socio-political context of the social determinants of health in many of the activities they provided (Table 2). Activities at this level revolved predominantly around Advocacy but also included activities related to Collaborations and Partnerships, Capacity Building and Community Empowerment, and Legal and Justice Services (Table 2). The following quote provides an example of a remote ACCHO responding to community need using a broad, multi-level approach. The ACCHO was working within the socio-political context through efforts to influence a Parliamentary Joint Committee as well as developing a responsive workforce and addressing the immediate health, social and cultural needs of their clients.

*“In response to the prevalence of crystalline methamphetamine use (ICE) and community concern, [we] advocated to strengthen existing AOD treatment services to provide access to medical care, psychological treatment and social and cultural support including intensive care management for all drug addictions including alcohol and ICE.’ [ACCHO] advocated against a separate, stand-alone service system for ICE users. We continued to argue for investment in early childhood as key to the prevention of all forms of addiction. [ACCHO] also submitted a response to the Parliamentary Joint Committee on Law Enforcement inquiry into ICE use. We commissioned [local training organisation] to deliver “Working with clients that use Methamphetamine (ICE)” Workshops for clinicians and social support staff, with over 60 staff attending.” [Remote ACCHO]*

ACCHO leaders and workforce also participate on committees that go beyond direct clinical or health-related conditions to broader public policy reflective of the broad social determinants of health. One example included an ACCHO improving communications through formal agreements between multiple agencies responsible for youth justice:

“*The [justice] program has now established communications with DHHS, Corrections and the Department of Justice and is also looking at developing MOUs with these agencies. [ACCHO] has been an active member of [Local Aboriginal Justice Advisory Committee] and a variety of [Aboriginal] Youth Justice Support networks and has been present to represent community members during court hearings.” [Inner Regional ACCHO]*

Within the reports, there is clear evidence of ACCHOs creating opportunities for Aboriginal societal and cultural values to be seen and expressed in the dominant western socio-political context in Australia.

*[ACCHO] has hosted Cultural Immersions for key external service partners and members of government, which has initiated culturally appropriate changes within their respective services and strengthened partnerships. This approach allows other organisations to hear from local women at a grass roots level, and initiate an interest and awareness of Aboriginal culture from a face to face experience, out on country. [Inner Regional ACCHO]*

### Socio-economic position

All 67 annual reports described ACCHO activities that aimed to reduce inequity related to socio-economic position. The socio-economic context was addressed by ACCHOs in every activity excluding clinical care (Table 2). This was demonstrated by ACCHOs providing support with and increasing access to social services (such as Centrelink, Housing, Human Services) through transport, advocacy, and case management. ACCHOs reported supporting families in the education of their children through a range of programs that promoted access to education, as demonstrated by the quotes below:

*[ACCHO] Indigenous Education Services: this program’s primary responsibility is to ensure increased school readiness for our young children between the age of three (3) to five (5). The program is being delivered to increase Aboriginal and Torres Strait Islander families and children’s smooth transition and success rate from Early Childhood into Prep, including contributing to community well-being. The service has also offered tutoring support and playgroups over the past 12 months. [Major City ACCHO]*

*Through casual conversation and genuine interest, [ACCHO worker] was able to identify her interests and, with another Case Manager, took [teenage girl] on a tour of a flexible learning centre which offered a flexible approach to schooling. [Major City ACCHO]*

Another example demonstrated how an ACCHO was able to provide career advice to support individuals’ employment prospects upon exiting a rehabilitation program:

*We ensure that we provide clients with support once they exit the rehabilitation centre by providing transitional housing and programs such as career advice counselling, training and assistance in obtaining ongoing independent accommodation for clients. [Major City ACCHO]*

Most ACCHOs reported a high proportion of Indigenous employment.

*Our clinic boasts 50% Indigenous staff, 100% female staff with 80% full time staff. [Major City ACCHO].*

Additionally, ACCHOs provided training opportunities for Indigenous staff to obtain qualifications through external channels and supported in-house training and development.

*[ACCHO] is proud to support two (2) [Aboriginal Health Workers] to undertake a Social Work degree through [a University] … .The [ACCHO] Research section continued to prioritise developing internal capacity through the ongoing employment of Aboriginal Research Officers (AROs) to develop select projects. [Remote ACCHO]*

Through partnerships ACCHOs contributed to reducing institutional racism. Not only was there considerable evidence that ACCHOs worked to build and maintain partnerships but there was also evidence that these partnerships created understanding of Aboriginal and Torres Strait Islander culture in mainstream organisations as demonstrated in the following quote:

*This project formed a working partnership with students and staff from [local private school] in the painting of a mural on the wall enclosing the garden that is demonstrative of community ownership and enhances the appearance bringing to mindfulness our connection to Country and ownership via participants’ hand prints and paintings on this wall. A second partnership with [NGO] resulted in the labour required and successful completion of Certificate II in Horticulture for 11 Participants who also received a certificate of appreciation for completing an eight week life skills program through [ACCHO]. [Outer Regional ACCHO]*

### Intermediary determinants

The intermediary determinants were addressed in all reported ACCHO activities (Table 2). While most commonly addressing clinical care, ACCHOs also engaged in intermediary determinants through extensive work in Community and Cultural engagement, Health Promotion, Drug, Alcohol and Addiction Services, and Mental Health (Table 2). ACCHOs demonstrated supporting their clients to navigate their way through health and social sectors to increase their access to these services.

Based on the reporting, ACCHOs understood the importance of material circumstance on health and wellbeing and intervened where they could, highlighted by one ACCHO that delivered on the need for food and basic household products for families in crisis,

*Family Services continued to manage the Food Bank here at [ACCHO], providing the community with fresh fruit, vegetables, pantry items and household products. Referrals for Emergency Relief to [Aboriginal Health Peak Body] for financial emergency assistance. Family Services have also been able to refer to other services as community has required it. With all the limitations on the program we are still able to have positive outcomes for families in crisis. [Major City ACCHO]*

One ACCHO identified the need to be able to provide specialised clinical care to Aboriginal and Torres Strait Islander people for conditions that are rarely seen in the mainstream Australian population,

*[Community health centre] Health Services has been proactive in addressing diseases and conditions which may be uncommon in a mainstream environment but are usually associated with extremes of poverty, such as rheumatic heart disease and trachoma, and which require specific expertise and services to address them. [Very Remote ACCHO]*

ACCHOs reported addressing the psychosocial health and wellbeing of many groups within the community, including men and women separately,

*We held a men’s painting group titled “Healing through Art” addressing cultural, spiritual and social wellbeing. Participants were encouraged to tell their story through art and express their ideas in a culturally safe environment and to discuss social, cultural and grief and loss issues with access to other supports relating to their wellbeing. [Major City ACCHO]*

*Women’s yarning groups; Have started in [local area], these groups are run at [community organisation] by the women with support from the counsellor. Discussions include but not limited to, grief and loss, trauma and, sexual abuse are discussed in relation to alcohol and other drug impacts. Also craft making and a nice lunch provided. [Remote ACCHO]*

### Emerging themes

#### Culture

Aboriginal and Torres Strait Islander culture and partnerships emerged as strong themes throughout the coded data. It was evident that culture was a major driver of service delivery and often standard clinical services had a cultural element embedded in their delivery. The quote below was chosen as an example of how an ACCHO demonstrated the importance of connection to Country by providing critical clinical care for their clients on-Country. The annual report explained,

*‘We are developing this service in response to strong community demand to be able to receive treatment on country and remain connected with family. It also allows for people to return home for short term respite stays to ensure they can fulfil important cultural and ceremony obligations. It is important to note that the community feel so strongly about this, that they have self-funded this program through royalty association money.’ [Very Remote ACCHO]*

The analysis revealed that through providing culturally safe care clients were more comfortable using the service. One Mums and Bubs program reported that clients prefer their culturally appropriate service:

*[ACCHO] Mums and Bubs provides a contemporary and modern approach to family medicine for Aboriginal and Torres Strait Islander families in the area. Traditional services such as ante-natal and post-natal care are provided in a personalised culturally appropriate service delivery environment. Our families feel safe and secure when visiting the service and prefer it when seeking support on lactation, immunisation, contraception advice and child health and development check ups. [Major City ACCHO]*

#### Social capital and social cohesion

In many of the services ACCHOs reported, there was evidence of efforts to strengthen or build social capital and social cohesion. The following example demonstrates the complexity of the work of ACCHOs in building partnerships with organisations improving their community’s social capital:

*The program currently has two staff members employed and provides support and cultural services to kindergartens which are listed as having Aboriginal children enrolled. There are currently approx. 25 kindergartens having had services being delivered with another one recently requesting service delivery. [ACCHO] works in close collaboration with the kindergarten teachers and cluster management organisations including [local kindergarten association] and [local council] to ensure that the services provided by [the ACCHO] meet both the needs of the community, children and Kindergarten teachers and also fall in alignment with Early Years policy framework, the [State Aboriginal Education plan] and the [Jurisdiction] Early Years Learning and Development Framework. [Inner Regional ACCHO]*

There was evidence of ACCHOs building strong partnerships with cross sector organisations so as to facilitate client access to services that could improve their living situation. The following quote demonstrates the work of an ACCHO to improve the social capital of their community through a partnership with a local housing organisation:

*“The strong collaboration and partnership between program staff and the Private Rental Liaison Officers situated within Housing [organisation], has seen a high number of clients obtain affordable housing in the private rental market.”*

*[Major City ACCHO]*

ACCHOs reported a range of community engagement activities that brought community together and promoted social cohesion, as described in the following examples.

*Regular community meetings and events were introduced so that all members and the broader community can meet with Board and management representatives face-to-face. This will be an ongoing program. [Remote ACCHO]*

*Youth Consultation Day at [youth community centre]– Our youth said they would enjoy opportunities to spend time with local elders, learn more about land and native plants and native art and dance. Key words that described ‘Culture’ for them: Native plants/animals, Dreamtime stories, Proud of who you are, Tradition, Dance (No opportunity for girls), NITV, Music (Modern/traditional), Food, ‘Knowing your mob’ and learning from family. [Outer Regional ACCHO]*

## Discussion

This is the first document analysis of ACCHO Annual Reports to describe ACCHO service provision addressing the social determinants of health and structural determinants of health inequity. It demonstrates that ACCHOs provide primary health care across the lifespan and frequently incorporate activities that address the social determinants of health and health inequity. As expected, all ACCHOs within the sample reported activities that addressed the intermediary determinants of health. Additionally, aligned with the NACCHO definition of health [11], all ACCHOs addressed the structural determinants of health inequity, specifically socioeconomic position and did so through all coded activities excepting clinical care. ACCHOs also worked to improve participation in education, providing financial services and delivering programs for personal empowerment, family support and community capacity building to better position clients to manage their health and social needs. More than half of all ACCHOs reported activities directed at improving the socio-political context of the structural determinants of health inequity which highlights the comprehensiveness of ACCHO service provision. These activities demonstrated the efforts of ACCHOs to improve the socio-political context for the community they served in the form of advocacy, collaborations and partnerships, and community empowerment and capacity building. This reach extended to addressing the socio-political context through specific programs such as housing and homelessness, mental health, drug and alcohol services and family support.

This culturally centred and comprehensive approach to primary health care by ACCHOs in Australia has similarities with Indigenous health programs internationally. Self-determination and the predominant medical focus of Indian Health Service (IHS) led the Navajo Tribal Council to establish the Navajo Division of Health in Arizona to deliver 15 programs that together address intermediary and structural determinants of health [22]. This was also the impetus of the Gila River Indian Tribe who through their Department of Public Health have contractual arrangements with IHS to deliver a broad range of programs relating to environmental health, food distribution, wellness and medical transport [22]. A program specific example of addressing sociocultural barriers with tele-ophthalmology was developed and delivered by the Alberta Cree community in Canada. They increased program participation by 65% by employing nurses from the local community fluent in Cree language, including religious and cultural artefacts in the screening protocols, and under the guidance of a spiritual leader who invited health into the participants through a ‘Smudge’ ceremony. In addition, open circle discussions focused on behavioural and psychosocial challenges and goal setting for healthy living [23]. In Mexico, the results of an evaluation of community-based health care for Indigenous women by Pelcastre-Villafuerte et al. found that,

‘*the processes of the Casas* [i.e. the Project] *transcends sexual and reproductive health care and cases of violence, and speaks to change within larger structural determinants of health equity. Implicitly, the work of the women who have participated in this project, from different places and at different moments, has contributed to strengthening and claiming of indigenous and gender equity, rooted in human rights’* [24]*.*

Similarities to ACCHOs in Australia extend beyond service and activity design and provision, to recognising the diverse needs and aspirations of their communities by embedding flexibility in how services are delivered, and activities are implemented. The prevailing medical paradigm has been identified as a factor hindering development of policy to address the social determinants of health in Australia [25]. ACCHOs are perhaps less constrained by the dominance of the medical paradigm since they have uniquely positioned themselves as a sector founded upon a holistic social health model that is governed by Aboriginal community members and actioned by teams of multidisciplinary health and community workers that include medical practitioners amongst, but not leading, their ranks. In addition, the population ACCHOs serve suffer overwhelming social inequity that is impossible to ignore or separate in the pursuit to provide culturally appropriate and adequate primary health care [15]. The evidence shows that ACCHOs provide the best returns on investment relating to primary health care access and quality, and add significant economic value to Aboriginal communities — indeed ACCHOs are a major employer of Aboriginal people [26] — and generate flow-on effects to other sectors [27]. From a policy perspective, it is recognised that for health equity to be reached, holistic and collaborative approaches by all sectors and mainstream services is needed with Aboriginal leadership and active involvement of Aboriginal communities in all stages of program delivery, implementation and evaluation [28].

Our study importantly showcases the extent of programs and activities undertaken by ACCHOs to strengthen culture and community engagement. There is widespread agreement that for Aboriginal and Torres Strait Islander peoples, health outcomes are greatly influenced by the ‘enabling, protecting and healing aspects’ of culture that are critical in fostering resilience and contributing to Indigenous identity (p.33) [29]. The cultural determinants of health promote resilience, foster identity and support holistic wellbeing and are promoted through ‘*traditional cultural practice, kinship, connection to land and Country, art, song and ceremony, dance, healing, spirituality, empowerment, ancestry, belonging and self-determination’* (p.7) [30]. It was evident throughout the analysis that culture is at the heart of ACCHO practice and was present throughout most activities described in annual reports which is not surprising given the importance of culture to the health and wellbeing of the community (p.9) [30]. This evidence is supported by findings of a 2018 systematic review where culture was identified as underpinning all ACCHO practice [31], and the National Aboriginal and Torres Strait Islander Health Plan 2013–2023 which places culture as the leading priority at the centre of change [30].

During our analysis we questioned the extent to which the WHO’s Conceptual Framework considered culture as a social determinant of health. Culture is integral to the health and wellbeing of Aboriginal and Torres Strait Islander people and Napier et al. argued that for all people “the systematic neglect of culture in health [is] the single biggest barrier to advancement of the highest attainable standard of health worldwide” (pg. 2) [32]. The WHO Social Determinants of Health Discussion Paper No. 2 does not define the concept of culture and societal values (socio-political context) or discuss their role as determinants of health and wellbeing. We therefore reconciled that culture and societal values that shape society represent those of the dominant population [2] rendering minority cultures and societal values invisible. This phenomenon could help explain the need for the strong presence of Aboriginal and Torres Strait Islander cultural expression in each ACCHO and across each of the determinants of health. Furthermore, we argue that culture and societal values, rather than being an independent socio-political factor, are the basis on which socio-political discourse and policies are built. Therefore, culture and societal values could be more accurately represented by placing greater weight on their influence on the social determinants of health and health equity. Since the publication of the framework, the literature on the relationship between culture and health and wellbeing has grown providing additional evidence to further develop the framework [6, 7, 32].

Social cohesion and social capital emerged as two major themes. Social cohesion and social capital are separate but interrelated phenomena that are important to promote health and address health inequities and are positioned specifically in the WHO Conceptual Framework to traverse intermediary and structural determinants [2]. Social cohesion refers to positive social relationships that form a cohesive society that can be described as one that “works towards the well-being of all its members, fights exclusion and marginalisation, creates a sense of belonging, promotes trust, and offers its members the opportunity of upward mobility”(p.17) [33]*.* Szreter and Woolcock 2004 [34], describe three types of social capital: bonding social capital; bridging social capital; and linking social capital. *Bonding social capital* refers to positive social relationships and trust between members of a similar social group that encourages reciprocity and exchange*. Bridging social capital* refers to mutual respect and cooperation between people and groups who are aware they are dissimilar in their socio-demographic characteristics. Lastly, *linking social capital* refers to respect and trust between individuals, groups and networks across formal, institutionalised lines of power and authority.

In some instances, ACCHOs intentionally promoted social cohesion through their programs. This work was evidenced in ACCHOs extensive efforts to promote community and cultural engagement through activities such as yarning groups, cultural healing camps, men’s sheds, and parenting groups. In other circumstances social cohesion was embedded in programs naturally, based on Aboriginal peoples’ values and understanding of health. Through strong kinship networks, Aboriginal cultures value interdependence and a collective rather than individual approach to living [35] which naturally promotes social cohesion and the social capital of individuals and communities. The need for programs that promote social cohesion may arise because the mechanisms for social cohesion were interrupted or broken due to colonisation. For example, racist policies such as the forcible removal of Aboriginal children from their families (p.178) [36], culture and connection to land were severed resulting in trauma spanning generations and disrupting existing social cohesion mechanisms within Aboriginal communities. The community-control model facilitates and is paramount [37] to the success of these types of programs and has the ability to respond to the diverse nature of Aboriginal communities where cultural practices and values may differ significantly from one community to another [38]. These activities work to reinforce *bonding social capital* within Aboriginal communities.

ACCHOs also worked to promote *bridging social capital*. ACCHOs occupy an important space to promote positive and respectful relationships between Indigenous and non-Indigenous people, organisations and communities. This was evidenced with their work in collaborations and partnerships, building cultural competency of the non-Indigenous workforce, and the hosting and/or participation in significant cultural events such as NAIDOC week, Reconciliation Week and Sorry Day. In addressing racism and cultural insensitivity in the community, such programs could reduce the structural determinants of health inequity endured by Aboriginal clients. It is important to recognise that within communities ACCHOs are not the only providers of services that address the social determinants of health. There are likely a number of organisations providing services, some of which are specifically funded to address the social determinants of health such as housing, education and employment. Given that these organisations already exist, ACCHOs efforts to strengthen bridging social capital is important as it links ACCHO clients to services that maybe difficult to access.

Finally, ACCHOs promoted *linking social capital* through advocacy work. This analysis found that ACCHOs are heavily engaged in advocacy activities to address the social and structural determinants of health. This demonstrates the collective actions undertaken by the sector to advocate for policy and programs to address health inequities for Aboriginal peoples at local, state and federal levels. Recommended policy actions in Australia to address the social determinants of health include targeted programs for disadvantaged groups, the closing of health gaps, and strategies to address the population-wide social gradient [2].

### Limitations

There are limitations to the approach used in this study. First, due to challenges in accessing annual reports from all ACCHOs our sample is not nationally representative, reflecting 47% of all ACCHOs. In addition to no representation of ACCHOs in one jurisdiction, the sample comprises a larger proportion of ACCHOs located in major cities (22%) compared to the total share of all ACCHOs (13%) and a smaller proportion compared to the total share of ACCHOs in outer regional locations (23% vs 41%). Furthermore, it has been well documented that ACCHOs experience a reporting overburden and given that the production of annual reports is not compulsory, this sample may bias those ACCHOs that had sufficient administrative resourcing. Second, reporting is not standardised across ACCHOs and therefore documentation of activities is inconsistent throughout annual reports and may not be comprehensive in both coverage of activities and their holistic nature. Third, in most cases it is unclear whether activities are linked to funding and as a result it is not possible to draw conclusions about the nature of ACCHO activities in comparison to funding. Although, given these limitations, the activities and the extent to which ACCHOs tackle the social determinants of health and health inequity is likely to be underestimated. An overarching limitation of not formally resourcing the role of ACCHOs in addressing the social determinants of health are the missed opportunities that result in compounding the disparities in health and social outcomes experienced by the Aboriginal and Torres Strait Islander population.

## Conclusion

This study provides evidence of the considerable efforts of the ACCHO sector, in addressing immediate healthcare needs and investing in advocating for and driving changes in the more challenging structural determinants of health equity. In particular, culture informs and is embedded in the services ACCHOs provide and through their work they promote social cohesion and social capital. ACCHOs should be recognised and appropriately supported for the instrumental and innovative work they do in improving the social determinants of health of Aboriginal and Torres Strait Islander populations.

The ACCHO sector provides a comprehensive model of primary health care that is inclusive of addressing the social determinants of health. Further research to understand the ability of the model and the level of investment required to interrupt the entrenched structural determinants that drive health inequity in Australia would be of great value to the population, the ACCHO sector and in informing wider adoption of the model.

## Supplementary Information

**Additional file 1.** Activities delivered by ACCHOs to specific population groups. The population groups that ACCHO delivered activities to.

## Data Availability

Annual reports were available online or by contacting the relevant organisation. In keeping with our ethics application, the organisations who were included in the study will remain confidential and therefore the annual reports have not been made available. The authors may be contacted for copies of the annual reports that were sourced on-line. Permission from the relevant organisation would need to be sought to obtain the annual report provided by the organisation, as ethical approval was only granted for the purpose of this study.

## Abbreviations

ACCHO: Aboriginal Community Controlled Health Organisation
IHS: Indian Health Service
NACCHO: National Aboriginal Community Controlled Health Organisation
OECD: Organisation for Economic Co-operation and Development
WHO: World Health Organisation
