# Supplementary material for: Aboriginal community controlled health organisations address health equity through action on the social determinants of health of Aboriginal and Torres Strait Islander peoples in Australia
Source: BMC Public Health. 2020 Dec 4;20:1859. doi: 10.1186/s12889-020-09943-4 (PMC7716440; doi:10.1186/s12889-020-09943-4)
Supplement: Supplementary file 1 — Additional file 1. Activities delivered by ACCHOs to specific population groups. The population groups that ACCHO delivered activities to. [file 12889_2020_9943_MOESM1_ESM.docx]

| ***ACTIVITIES*** | **Children and Young People*** | **Mothers and Babies** | **Women** | **Men** | **Older People** | **Families** | **People with Disability** | **People in the justice system** | **LGBTQI** |
| --- | --- | --- | --- | --- | --- | --- | --- | --- | --- |
| **HEALTH PROMOTION AND EDUCATION** | 50 | 26 | 25 | 22 | 10 | 7 | 1 | 1 | 0 |
| **CLINICAL CARE** | 42 | 36 | 21 | 12 | 10 | 2 | 2 | 1 | 0 |
| **COMMUNITY AND CULTURAL ENGAGEMENT** | 35 | 14 | 31 | 26 | 27 | 4 | 6 | 7 | 1 |
| **SCHOOLS, EDUCATION AND TRAINING** | 35 | 9 | 7 | 8 | 3 | 2 | 0 | 4 | 0 |
| **FAMILY SUPPORT** | 23 | 20 | 11 | 9 | 1 | 23 | 0 | 3 | 0 |
| **MENTAL HEALTH** | 23 | 8 | 3 | 3 | 1 | 3 | 1 | 1 | 0 |
| **PERSONAL EMPOWERMENT** | 18 | 1 | 3 | 1 | 3 | 2 | 1 | 1 | 0 |
| **DRUG, ALCOHOL AND ADDICTION** | 16 | 12 | 9 | 18 | 3 | 6 | 0 | 9 | 0 |
| **LEGAL AND JUSTICE SERVICES** | 12 | 0 | 0 | 2 | 0 | 3 | 0 | 17 | 0 |
| **TRANSPORT** | 7 | 5 | 4 | 1 | 1 | 5 | 3 | 0 | 0 |
| **CASE MANAGEMENT** | 6 | 6 | 1 | 0 | 9 | 1 | 3 | 1 | 0 |
| **ADVOCACY** | 6 | 0 | 0 | 3 | 1 | 1 | 0 | 5 | 0 |
| **HOUSING AND HOMELESSNESS** | 4 | 3 | 5 | 8 | 1 | 2 | 1 | 3 | 0 |
| **CAPACITY BUILDING AND COMMUNITY EMPOWERMENT** | 3 | 1 | 2 | 2 | 2 | 1 | 2 | 0 | 0 |
| **FINANCIAL SERVICES** | 3 | 1 | 0 | 0 | 1 | 2 | 0 | 1 | 0 |
| **HACC OR CHSP^(b)^** | 2 | 0 | 1 | 1 | 17 | 0 | 7 | 0 | 0 |
| **EMPLOYMENT** | 0 | 1 | 1 | 1 | 0 | 0 | 0 | 0 | 0 |
